# Involvement of TAGE-RAGE System in the Pathogenesis of Diabetic Retinopathy

**DOI:** 10.1155/2010/170393

**Published:** 2010-06-22

**Authors:** Masayoshi Takeuchi, Jun-ichi Takino, Sho-ichi Yamagishi

**Affiliations:** ^1^Department of Pathophysiological Science, Faculty of Pharmaceutical Sciences, Hokuriku University, Ho-3 Kanagawa-machi, Kanazawa 920-1181, Japan; ^2^Department of Pathophysiology and Therapeutics of Diabetic Vascular Complications, Kurume University School of Medicine, 67 Asahimachi, Kurume 830-0011, Japan

## Abstract

Diabetic complications are a leading cause of acquired blindness, end-stage renal failure, and accelerated atherosclerosis, which are associated with the disabilities and high mortality rates seen in diabetic patients. Continuous hyperglycemia is involved in the pathogenesis of diabetic micro- and macrovascular complications *via* various metabolic pathways, and numerous hyperglycemia-induced metabolic and hemodynamic conditions exist, including increased generation of various types of advanced glycation end-products (AGEs). Recently, we demonstrated that glyceraldehyde-derived AGEs, the predominant structure of toxic AGEs (TAGE), play an important role in the pathogenesis of angiopathy in diabetic patients. Moreover, recent evidence suggests that the interaction of TAGE with the receptor for AGEs (RAGE) elicits oxidative stress generation in numerous types of cells, all of which may contribute to the pathological changes observed in diabetic complications. In this paper, we discuss the pathophysiological role of the TAGE-RAGE system in the development and progression of diabetic retinopathy.

## 1. Introduction

Diabetic complications are a leading cause of end-stage renal failure, acquired blindness, and cardiovascular disease (CVD) and are involved in the disabilities and high mortality rates observed in patients with type 1 or type 2 diabetes [1]. Although various hyperglycemia-induced metabolic and hemodynamic conditions are proposed to contribute to complications in diabetes [2, 3], recent clinical studies have suggested the concept of “hyperglycemic memory” in the pathogenesis of vascular injury in diabetes [4–6]. Indeed, the Diabetes Control and Complications Trial-Epidemiology of Diabetes Interventions and Complications (DCCT-EDIC) Study demonstrated that the reduction in the risk of progressive retinopathy and nephropathy brought about by intensive therapy in patients with type 1 diabetes persisted for at least eight years, despite increasing hyperglycemia [4, 5]. The intensive therapy administered during the DCCT resulted in decreased progression of intima media thickness (IMT) and had reduced the risk of nonfatal myocardial infarction, stroke, or death from CVD by 57% by 11 years after the end of the trial [6]. 

Furthermore, a recent follow-up study, the United Kingdom Prospective Diabetes Study (UKPDS), has also shown that the benefits of intensive therapy in patients with type 2 diabetes were sustained after the cessation of the trial [7]. In this study, despite the early loss of glycemic differences between intensive and conventional therapy, the reductions in microvascular risk and emergent risk reductions for myocardial infarction and death from any cause were maintained during 10 years of posttrial follow-up [7]. These observations indicate that intensive therapy to control blood glucose has long-term beneficial effects on the risk of diabetic retinopathy, nephropathy, CVD, and death in patients with type 1 or type 2 diabetes, strongly suggesting that so-called “metabolic memory” causes chronic damage in diabetic vessels that is not easily reversed, even by subsequent, relatively good control of blood glucose. Among the various pathways activated under diabetes, as described above, the biochemical nature of advanced glycation end-products (AGEs) and their mode of action are the most compatible with the theory of “hyperglycemic memory” [8, 9]. 

 There is a growing body of evidence to suggest that continuous hyperglycemia under diabetic conditions enhances the formation of AGEs, senescent macroprotein derivatives, through nonenzymatic glycation (called the “Maillard reaction”). There is also accumulating evidence that the binding of the receptor for AGEs (RAGE) with AGEs elicits oxidative stress generation and subsequently evokes inflammatory and/or thrombogenic responses in various types of cells, thus participating in the development and progression of diabetic angiopathies [10–18]. Recently, we demonstrated that glyceraldehyde-derived AGEs (Glycer-AGEs), the predominant structure of toxic AGEs (TAGE), play an important role in the pathogenesis of angiopathy in diabetic patients [10, 19, 20]. Furthermore, there is a growing body of evidence to suggest that the interaction of TAGE with the RAGE alters intracellular signaling, gene expression, and the release of proinflammatory molecules and elicits oxidative stress generation in numerous types of cells, all of which may contribute to the pathological changes seen in diabetic complications. Therefore, the inhibition of TAGE formation, blockade of TAGE-RAGE interactions, and the suppression of RAGE expression or its downstream pathways are promising targets for therapeutic intervention against diabetic complications.

In this paper, we discuss the pathophysiological role of the TAGE-RAGE-oxidative stress system in the development and progression of diabetic retinopathy and related therapeutic interventions. 

## 2. Alternative Routes for the Formation of AGEs In Vivo

 AGEs are formed by the Maillard process, a nonenzymatic reaction between aldehyde or ketone group of the reducing sugars (such as glucose, fructose, and trioses etc.) and the amino groups of proteins that contribute to the aging of proteins and to the pathological complications of diabetes [10–13, 19–24]. In the hyperglycemia elicited by diabetes, this process begins with the conversion of reversible Schiff base adducts to more stable, covalently bound Amadori rearrangement products. Over the course of days to weeks, these Amadori products undergo further rearrangement reactions to form irreversibly bound moieties known as AGEs. AGEs were originally characterized by their yellow-brown fluorescent color and their ability to form cross-links with and between amino groups, but the term is now used for a broad range of advanced products of the glycation process, including *N*-(carboxymethyl)lysine (CML) and pyrraline, which show neither color nor fluorescence and are not cross-linked proteins [8, 21–25]. The formation of AGEs in vivo is dependent on the turnover of the chemically modified target, the time available, and the sugar concentration. The structures of the various cross-linked AGEs that are generated in vivo have not yet been completely determined. Due to their heterogeneity and the complexity of the chemical reactions involved, only some AGEs have been structurally characterized in vivo. The structural identities of AGEs with cytotoxic properties therefore remain unknown.

**Figure 1:**
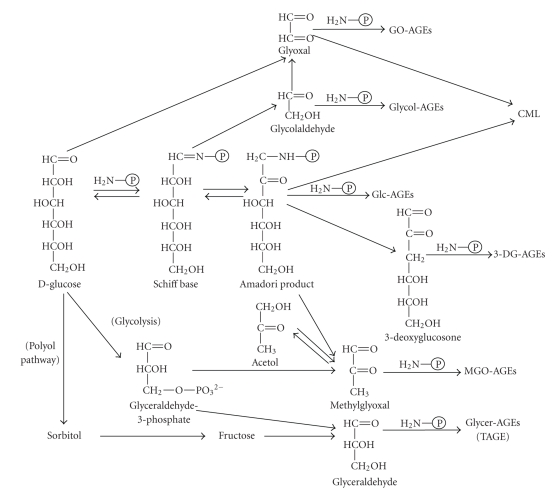
*Alternative routes for the formation of various distinct AGEs in vivo*. Glc-AGEs; glucose-derived AGEs, Glycer-AGEs; glyceraldehyde-derived AGEs, Glycol-AGEs; glycolaldehyde-derived AGEs, MGO-AGEs; methylglyoxal (MGO)-derived AGEs, GO-AGEs; glyoxal (GO)-derived AGEs, and 3-DG-AGEs, 3-deoxyglucosone (3-DG)-derived AGEs, CML; *N*-(carboxymethyl)lysine, and P-NH_2_; free amino residue of protein.

 Recent studies have suggested that AGEs can arise not only from reducing sugars, but also from carbonyl compounds derived from the autoxidation of sugars and other metabolic pathways [26–28]. Indeed, we have recently demonstrated that glucose, *α*-hydroxyaldehydes (glyceraldehyde and glycolaldehyde), and dicarbonyl compounds (methylglyoxal; MGO, glyoxal; GO, and 3-deoxyglucosone, 3-DG) are actively involved in the protein glycation process [21, 29–31]. Six immunochemically distinct classes of AGEs (glucose-derived AGEs; Glc-AGEs, glyceraldehyde-derived AGEs; Glycer-AGEs, glycolaldehyde-derived AGEs; Glycol-AGEs, MGO-derived AGEs; MGO-AGEs, GO-derived AGEs; GO-AGEs, and 3-DG-derived AGEs; 3-DG-AGEs) are found in the sera of type 2 diabetic patients during hemodialysis [21, 29–31]. Based on these data, we proposed a pathway for the in vivo formation of distinct AGEs involving the Maillard reaction, sugar autoxidation, and sugar metabolic pathways, as shown in Figure 1.

## 3. Receptors for AGEs

 Such receptors may play a critical role in AGEs-related biology and the pathology associated with diabetic complications and aging disorders. Several types of AGEs binding proteins and/or receptors for AGEs such as RAGE [32–36]; oligosaccharyl transferase-48 (AGE-R1) [37]; galectin-3 (AGE-R3) [38]; CD36 [39]; macrophage scavenger receptors types 1 and 2 (MSRs-1 & -2) [40]; and fasciclin EGF-like, laminin-type EGF-like, and link domain-containing scavenger receptors 1 and 2 (FEELs-1 & -2) [41] have been reported. The relative pathogenic contributions of these receptors to diabetic complications are poorly defined, although RAGE is by far the best characterized, and mechanistic in vitro and in vivo studies on AGEs and their regulatory fragments such as soluble RAGE (sRAGE) have indicated that they play important roles in pathobiology [36, 42]. RAGE is normally expressed in a variety of cells, including endothelial cells (EC), pericytes, neurons, and microglia, [32–34]. We have recently found that glyceraldehyde rapidly reacts with the amino groups of proteins to form Glycer-AGEs both in vitro and in vivo [19, 21, 30]. Furthermore, Glycer-AGEs have the strongest binding affinity for RAGE and subsequently elicit oxidative stress generation and vascular inflammation and are therefore implicated in accelerated atherosclerosis in diabetes [43, 44]. Recently, we also demonstrated that Glycer-AGEs, the predominant structure of toxic AGEs (TAGE), play an important role in the pathogenesis of angiopathy in diabetic patients [19, 20]. Moreover, there is a growing body of evidence to suggest that the interaction of TAGE with RAGE elicits oxidative stress generation in numerous types of cells, all of which may contribute to the pathological changes observed in diabetic complications [10, 19, 20].

## 4. Pathway of Glycer-AGEs (TAGE) Formation In Vivo

**Figure 2:**
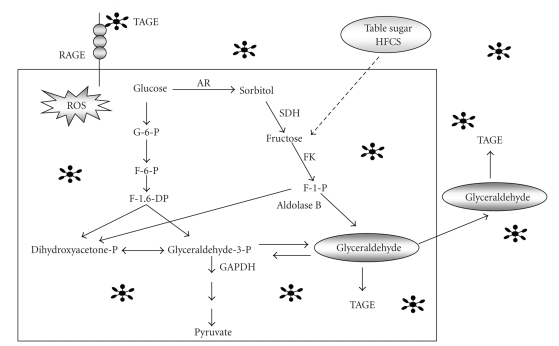
*Production routes of glyceraldehyde-derived AGEs (Glycer-AGEs) in vivo*. TAGE; toxic AGEs (glyceraldehyde-derived AGEs), RAGE; receptor for AGEs, ROS; reactive oxygen species, HFCS; high-fructose corn syrup, AR; aldose reductase, SDH; sorbitol dehydrogenase, FK; fructokinase, GAPDH; glyceraldehyde-3-phosphate dehydrogenase, ∗; TAGE.

Glyceraldehyde is derived from two distinct pathways in vivo, (1) the glycolytic pathway and (2) the fructose metabolism pathway [19, 20, 45]. (1) The glycolytic intermediate glyceraldehyde-3-phosphate (G-3-P) is normally catabolized by the enzyme glyceraldehyde-3-phosphate dehydrogenase (GAPDH). With a decline in GAPDH activity, G-3-P accumulates intracellularly. G-3-P metabolism then shifts to another route, and the amount of glyceraldehyde is increased, which leads to an increase in the formation of TAGE. This suggests a positive feedback mechanism; that is, the TAGE-induced GAPDH suppression further stimulates the generation of TAGE. (2) Under hyperglycemic conditions, the increased intracellular glucose concentration stimulates the polyol pathway to generate fructose in insulin-independent tissues such as the lens, kidney, nerve tissue, brain, and red blood cells [46–48]. Furthermore, fructose, a component of table sugar and high-fructose corn syrup, is also obtained from the diet [49]. Fructose is phosphorylated to fructose-1-phosphate (F-1-P) and then catabolized to glyceraldehyde and dihydroxyacetone-phosphate by aldolase B [48–51]. The newly synthesized glyceraldehyde is then transported or leaks passively across the plasma membrane. Glyceraldehyde promotes the formaion of TAGE both intracellularly and extracellularly (Figure 2).

## 5. Diabetic Retinopathy

 Diabetic retinopathy is one of the most important microvascular complications in diabetes and is a leading cause of acquired blindness among people of occupational age [52]. Hyperglycemia damages retinal microvascular cells and causes various changes in retinal tissues such as enhanced vascular permeability due to pericyte loss, which is followed by microvascular occlusion in the retina [53, 54]. Pericytes are elongated cells of mesodermal origin, which wrap around and along the EC of small vessels [55]. As pericytes contain contractile muscle filaments on their EC side, they are regarded as microvascular counterparts of smooth muscle cells and are considered to be involved in the maintenance of capillary tone [56, 57]. AGEs have been postulated to play a role in the development and progression of microvascular disease in diabetes. Vascular endothelial growth factor (VEGF) is a specific mitogen to EC, which is also known as vascular permeability factor and is generally thought to be involved in the pathogenesis of proliferative diabetic retinopathy. Indeed, clinical observations have demonstrated that the VEGF level in ocular fluid is positively correlated with the amount of neovascularization in diabetic retinopathy [58, 59]. 

 Retinal pericytes accumulate AGEs during diabetes [60], which is expected to have a detrimental influence on pericyte survival and function [61]. We have found that TAGE causes the apoptosis of retinal pericytes and induces the expression of VEGF by interacting with RAGE, indicating the involvement of TAGE in the pathogenesis of diabetic retinopathy, especially in the early stage [62–64]. TAGE also induces VEGF expression, DNA synthesis, and angiogenesis in EC. These changes are the hallmark of proliferative diabetic retinopathy [65, 66]. These findings suggest that the TAGE-RAGE interaction facilitates angiogenesis by two distinct mechanisms, by relieving the restriction on EC growth due to the apoptotic cell death of pericytes and by autocrine and paracrine induction of VEGF proteins by vascular wall cells. Although the molecular mechanisms of the VEGF overexpression elicited by TAGE are not fully understood, our recent investigation suggested that the TAGE-RAGE interaction increases VEGF gene transcription in EC by NADPH oxidase-mediated reactive oxygen species (ROS) generation and the subsequent activation of nuclear factor *κ*B (NF-*κ*B) *via* the Ras-mitogen activated protein kinase pathway [65, 66]. There has been increasing interest in the role of inflammatory reaction in diabetic retinopathy [67]. AGEs have recently been shown to increase leukocyte adhesion to cultured retinal microvascular EC by inducing intracellular cell adhesion molecule-1 (ICAM-1) expression [68]. Furthermore, TAGE also induces monocyte chemoattractant protein-1 (MCP-1) expression in EC through intracellular ROS generation [69]. 

## 6. Postprandial Hyperglycemia is Associated with Increased Risk of Diabetic Retinopathy

 While it is well known that postchallenge and postprandial hyperglycemia are related to the development and progression of diabetic macrovascular disease [70, 71], there are limited data on the relationship between postprandial hyperglycemia and microvascular complications. A recent observational prospective study from Japan demonstrated that postprandial hyperglycemia is a better predictor of diabetic retinopathy that glycated hemoglobin A_1c_ (HbA_1c_) [72]. Shiraiwa et al. performed a cross-sectional study of 232 people with type 2 diabetes who were not being treated with insulin injections. Multiple regression analysis revealed that postprandial hyperglycemia was independently correlated with the incidence of diabetic retinopathy and neuropathy. Additionally, postprandial hyperglycemia was also found to be associated, although not independently, with the incidence of diabetic nephropathy.

 We have previously shown that glyceraldehyde reacts rapidly with the amino groups of proteins to form TAGE in vivo, which evokes vascular inflammation and oxidative stress generation, thereby implicating them in accelerated atherosclerosis in diabetes [10, 19, 20]. More recently, we investigated the effects of nateglinide, which has been known to improve postprandial hyperglycemia, on HbA_1c_, Glc-AGE, and TAGE levels in Goto-Kakizaki (GK) rats, one of the rat models of type 2 diabetes, fed twice a day [73]. After 6 weeks, nateglinide treatment was found to not only prevent postprandial hyperglycemia, but also to reduce TAGE levels in GK rats. However, it did not cause a significant difference in HbA_1c_ or Glc-AGE levels [73]. This study suggests that TAGE is formed more rapidly than HbA_1c_, a precursor of Glc-AGEs, under postprandial hyperglycemic states and shows potential as novel markers of cumulative postprandial hyperglycemia. In this study, although we did not clarify the exact molecular mechanism by which TAGE is formed under postprandial hyperglycemic conditions, hyperglycemia-induced oxidative stress-mediated inhibition of GAPDH may lead to the elevation of glyceraldehyde levels and subsequently enhance the formation of TAGE during the postprandial period [74]. The relative contribution of postprandial glucose decreased progressively from the lowest to the highest quintile of HbA_1c_; whereas, the relative contribution of fasting glucose increased gradually with increasing levels of HbA_1c_ [75]. These observations suggest that a decrease in HbA_1c_ levels does not necessarily reflect a reduction in postprandial hyperglycemia, especially in poorly controlled diabetic patients.

## 7. Serum Levels of TAGE in Diabetes

 The above-discussed effect of TAGE strongly suggests a pathological role for these senescent macroproteins in diabetic complications. Furthermore, Glc-AGEs and TAGE are present in human serum, and the level of both AGEs is elevated in type 1 and type 2 diabetes [76–79]. These AGEs, especially TAGE-epitopes, elicit angiogenesis at the concentrations present in the sera of diabetic patients. These results suggest the involvement of TAGE-epitopes in pathologic angiogenesis in vivo. Recently, we demonstrated that the vitreous levels of both TAGE and VEGF were significantly higher in diabetic patients than in control subjects and that these levels were elevated in association with the severity of neovascularization in diabetic retinopathy. In addition, there was a significant correlation between vitreous TAGE and VEGF levels [80, 81]. Furthermore, we have recently found that serum TAGE levels are positively correlated with thrombogenic markers in humans. Plasminogen activator inhibitor-1 and fibrinogen levels are positively associated with serum TAGE levels [82].

 While many of the reported studies measured a range of ill-defined AGEs moieties, others evaluated defined adducts such as CML, pentosidine, and crossline in association with diabetic retinopathy [83, 84]. In addition, other studies have reported no correlation between AGE levels and retinopathy in diabetic patients [83, 85], although the apparent disparity between the findings of various studies may be related to variations in patient populations and/or the nonuniform assays used for plasma AGEs-quantification. Our studies suggest that an elevated TAGE level in diabetic patients is an important factor for the initiation and progression of retinopathy. Therefore, the inhibition of TAGE formation and the blockade of TAGE-RAGE interactions are potential therapeutic strategies for the prevention of diabetic retinopathy.

## 8. Serum Levels of Soluble RAGE in Diabetes

 The administration of a recombinant soluble form of RAGE (sRAGE) consisting of its extracellular ligand-binding domain has recently been shown to not only suppress the development of atherosclerosis but also to stabilize established atherosclerosis in diabetic apolipoprotein E-null mice [86, 87]. The blockade of the AGEs-RAGE axis by the administration of sRAGE also ameliorates neuronal dysfunction and reduces the development of acellular capillaries and pericyte ghosts in hyperglycemic and hyperlipidemic mice [88]. Furthermore, Kaji et al. have also shown that attenuation of the RAGE axis with sRAGE inhibits retinal leukostasis and blood-retinal barrier breakdown in diabetic C57/BJ6 and RAGE-transgenic mice, which are accompanied by decreased expression of VEGF and ICAM-1 in the retina [89]. These observations suggest that exogenously administered sRAGE captures and eliminates circulating AGEs, thus protecting against AGEs-elicited tissue damage by acting as a decoy. 

 Recently, endogenous sRAGE has been identified in humans [42]. Endogenous sRAGE may be generated from the cleavage of cell surface full-length RAGE or novel splice variants of RAGE (the C-truncated splice isoform of secretory RAGE; esRAGE) [42]. Endogenous total sRAGE levels are elevated in patients with type 1 or 2 diabetes [90–93]. Furthermore, we, along with others, have recently demonstrated that serum total sRAGE levels are positively, rather than inversely, associated with TAGE levels in both nondiabetic and diabetic subjects [93, 94]. Age-, sex-, and body mass index-adjusted TAGE levels are also significantly increased in proportion to the increasing levels of sRAGE in nondiabetic subjects [93, 94]. These findings suggest that the sRAGE pool is not able to efficiently capture and eliminate circulating TAGE in vivo by working as a decoy receptor. Since TAGE is a positive regulator of the cell expression of RAGE, circulating sRAGE levels may reflect tissue RAGE expression and be elevated in parallel with serum TAGE levels as a counter system against TAGE-elicited tissue damage [95–98]. 

 The serum levels of esRAGE are also correlated with the levels of circulating AGEs such as CML and pentosidine in type 1 diabetes [99]. However, in contrast to the case for total sRAGE, circulating esRAGE levels are decreased, rather than increased, in both type 1 and 2 diabetes. Katakami et al. reported in Japanese that the serum levels of esRAGE were significantly decreased in patients with type 1 diabetes compared with nondiabetic subjects [100], and esRAGE levels were found to be significantly lower in type 1 diabetic patients with retinopathy than in those without retinopathy [100, 101]. Decreased esRAGE levels were also found to be an independent risk factor for carotid atherosclerosis [102]. Indeed, Koyama et al. reported that esRAGE levels were decreased in Japanese type 2 diabetic patients compared with nondiabetic subjects and that low levels of esRAGE were associated with the components of metabolic syndrome and carotid atherosclerosis [102]. These observations were contrary to the finding of previous reports that total sRAGE levels were associated with conventional coronary risk factors including inflammatory markers and were independent determinants of coronary artery disease in diabetes [91, 95, 96]. Therefore, the kinetics and role of sRAGE and esRAGE in diabetes may differ [97]. Decreased levels of esRAGE may be associated with comorbidities such as diabetic retinopathy and atherosclerosis *via* mechanisms other than its role as a decoy because esRAGE levels are approximately 3~4-fold lower than total sRAGE levels and may not be sufficient to efficiently eliminate circulating AGEs in humans. Furthermore, sRAGE, but not esRAGE, was recently found to be independently correlated with albuminuria in type 2 diabetic patients [103].

## 9. Agents That Could Potentially Suppress TAGE-RAGE Interaction

### 9.1. Inhibitors of the Renin-Angiotensin System (RAS)

 The interaction of the RAS and TAGE-RAGE systems has also been proposed. We have found that angiotensin II potentiates the deleterious effects of TAGE in pericytes by inducing RAGE protein expression [64]. In vivo, TAGE-injection stimulated RAGE expression in the eyes of spontaneously hypertensive rats, which was blocked by telmisartan. In vitro, angiotensin II-type 1 receptor-mediated ROS generation elicited RAGE gene expression in retinal pericytes through NF-*κ*B activation. Furthermore, angiotensin II augmented TAGE-induced pericyte apoptosis, the earliest hallmark of diabetic retinopathy. Telmisartan also blocks angiotensin II-induced RAGE expression in EC [104].

 There is an increasing interest in the role of inflammatory reactions and immune phenomena in the pathogenesis of diabetic complications [105–107]. Indeed, leukocyte adhesion to diabetic retinal vasculature is considered to be a critical early event in diabetic retinopathy, the development of which is mainly mediated by VEGF, ICAM-1, and MCP-1 expression [105–107]. ICAM-1 and MCP-1 are essential chemokines that mediate the recruitment of leukocytes to mesangial lesions [108, 109]. The selective targeting of ICAM-1 or MCP-1 was also shown to markedly decrease albuminuria and renal injury in experimental diabetic nephropathy [108, 109]. Furthermore, several experimental studies have supported the pathological role of VEGF in diabetic nephropathy: antibodies raised against VEGF have been reported to improve hyperfiltration and albuminuria in diabetic rats [110, 111]. In addition, atherosclerosis is also an inflammatory-proliferative disease [112], and the administration of VEGF is reported to enhance atherosclerotic plaque progression in animals [113]. We have recently found that treatment with telmisartan or olmesartan inhibits the TAGE-evoked inflammatory responses in EC *via* downregulation of RAGE expression [114–118]. These observations suggest that the blockade of the TAGE-RAGE signaling pathways by RAS inhibitors may be clinically relevant to the prevention of diabetic complications.

### 9.2. Pigment-Epithelium-Derived Factor (PEDF)

 PEDF is a glycoprotein that belongs to a superfamily of serine protease inhibitors with complex neurotrophic, neuroprotective, antiangiogenic, antioxidative, and anti-inflammatory properties, any of which could potentially be exploited as a therapeutic option for the treatment of vascular complications in diabetes [119, 120]. PEDF inhibits TAGE-induced ROS generation and subsequently prevents apoptotic cell death in pericytes by restoring downregulation of the gene expression of the antiapoptotic factor bcl-2 [121]. Furthermore, PEDF also inhibits TAGE-induced ICAM-1, VEGF, and MCP-1 upregulation as well as NO suppression in EC by blocking NADPH oxidase-mediated ROS generation [69, 122–126]. In vivo, the administration of PEDF or pyridoxal phosphate, an AGEs inhibitor, decreased the retinal levels of 8-hydroxydeoxyguanosine (8-OHdG), an oxidative stress marker, and subsequently suppressed ICAM-1 gene expression and retinal leukostasis in diabetic rats [127]. Moreover, intravenous administration of TAGE to normal rats increased ICAM-1 gene expression and retinal leukostasis, which were blocked by PEDF [127]. PEDF inhibited diabetes- or TAGE-induced RAGE gene expression by blocking superoxide-mediated NF-*κ*B activation [128]. In addition, we have recently found that intravenous administration of TAGE to normal rats not only increases retinal vascular permeability by stimulating VEGF expression, but also decreases retinal PEDF levels [129]. Simultaneous treatment with PEDF inhibited TAGE-elicited VEGF-mediated permeability by downregulating the mRNA levels of p22^phox^ and gp91^phox^, membrane components of NADPH oxidase, and subsequently decreasing retinal levels of the oxidative stress marker, 8-OHdG. PEDF also inhibited TAGE-induced vascular hyperpermeability (as measured by transendothelial electrical resistance) by suppressing VEGF expression. PEDF decreased ROS generation in TAGE-exposed EC by suppressing NADPH oxidase activity *via* downregulation of the mRNA levels of p22^phox^ and gp91^phox^. This led to blockade of TAGE-elicited Ras activation and NF-*κ*B-dependent VEGF gene induction in EC. These results indicate that the central mechanism of PEDF inhibition of the TAGE-signaling related to vascular permeability is the suppression of NADPH oxidase-mediated ROS generation and subsequent VEGF expression [129]. 

 The PEDF levels in the aqueous humor and vitreous are decreased in diabetic patients, especially in those with proliferative retinopathy, suggesting that loss of PEDF in the eye contributes to the pathogenesis of proliferative diabetic retinopathy [130, 131]. We have also found that the vitreous levels of TAGE and VEGF are significantly higher in diabetic patients than in control subjects [81] and detected a significant correlation between vitreous TAGE and VEGF levels. Total antioxidant status was also decreased in the vitreous in patients with diabetes compared with that in the controls. Furthermore, both the TAGE and VEGF levels (inversely) and those of PEDF (positively) were associated with the total antioxidant status of the vitreous [132, 133]. These observations further support the concept that PEDF is an endogenous anti-inflammatory and antioxidative agent that blocks the TAGE-VEGF axis, thereby protecting against the progression of diabetic retinopathy.

### 9.3. Statins and Bisphosphonates

 We have found that protein prenylation is crucial for TAGE-RAGE signaling in EC [65, 66]. Cerivastain completely prevented TAGE-induced increases in NF-*κ*B activity and VEGF expression and the resultant increase in DNA synthesis as well as tube formation in microvascular EC [65]. Since mevalonate blocked the growth-inhibitory effects of cerivastatin on TAGE-exposed EC and that FTI-276, an inhibitor of farnesyltransferase, mimicked the effects of cerivastatin, cerivastatin may block the TAGE-RAGE signaling involved in vascular hyperpermeability and angiogenesis *via* the suppression of protein prenylation. Furthermore, we have recently found that atorvastatin dose-dependently inhibited TAGE-induced ROS generation in Hep3B cells [134]. Atorvastatin as well as the antioxidant N-acetylcysteine (NAC) was found to suppress C-reactive protein (CRP) expression in TAGE-exposed Hep3B cells at both the mRNA and protein levels [134]. These results demonstrate that atorvastatin is able to block the TAGE-signaling involved in CRP expression through its antioxidative action. Taken together, these observations suggest that statins have vasculoprotective effects by inhibiting the deleterious effects of TAGE *via* the suppression of their downstream signaling.

 Bisphosphonates are potent inhibitors of bone resorption and are widely used for the treatment of osteoporosis, osteolytic bone metastasis, and tumor-associated hypercalcemia [135–137]. These compounds have a high affinity for calcium ions and therefore target bone mineral, where they are internalized by bone-resorbing osteoclasts and inhibit osteoclast function. Recently, farnesyl pyrophosphate synthase has been shown to be a molecular target of nitrogen-containing bisphosphonates such as incardronate disodium and minodronate, and the inhibition of the posttranslational prenylation of small molecular weight G proteins including Ras and Rac-1 is probably involved in their antiresorptive activity in osteoclasts [135–137]. Since the protein prenylation of GTP-binding proteins is associated with various cellular functions such as cell growth and differentiation [135–137], nitrogen-containing bisphosphonates may have pleitrophic effects by blocking the synthesis of isoprenoid intermediates. Indeed, incardronate disodium was found to inhibit TAGE-induced increases in NF-*κ*B activity and VEGF expression as well as the proliferation and tube formation of EC [66]. Furthermore, we have recently found that minodronate inhibits TAGE–induced NF-*κ*B activation and subsequently suppresses VCAM-1 gene expression by reducing ROS generation in EC [135]. Geranylgeranyl pyrophosphate reversed the antioxidative properties of minodronate in TAGE-exposed EC [135]. Taken together, these findings suggest that nitrogen-containing bisphosphonates are able to inhibit TAGE-elicited inflammatory-proliferative changes in EC by suppressing NADPH oxidase-derived ROS generation, probably *via* the inhibition of the geranylgeranylation of Rac-1, a component of endothelial NADPH oxidase [136, 137]. 

## 10. Conclusion

There is accumulating evidence that the TAGE-RAGE-oxidative stress system is actively involved in the pathogenesis of diabetic complications, especially diabetic retinopathy. We have reviewed the inhibitors of the TAGE-RAGE axis and their potential therapeutic implications in these devastating disorders.

**Expert Opinion**

Two recent large prospective clinical studies, DCCT and UKPDS, have shown that intensive blood glucose control effectively reduces the incidence of vascular complications among patients with diabetes [138, 139]. However, strict control of hyperglycemia is often very difficult to maintain and may increase the risk of severe hypoglycemia in diabetic patients. Inhibition of TAGE formation, blockade of TAGE-RAGE interactions, and the suppression of RAGE expression or its downstream pathways by the agents discussed here are promising novel therapeutic strategies for the treatment of patients with diabetic retinopathy. Further clinical studies are needed to clarify whether the use of these agents is able to reduce the risk of diabetic retinopathy beyond blood glucose-, blood pressure- or cholesterol-lowering effects.

## References

[1] Laakso M (1999). Hyperglycemia and cardiovascular disease in type 2 diabetes. *Diabetes*.

[2] Nishikawa T, Edelstein D, Du XL (2000). Normalizing mitochondrial superoxide production blocks three pathways of hyperglycaemic damage. *Nature*.

[3] Brownlee M (2001). Biochemistry and molecular cell biology of diabetic complications. *Nature*.

[4] Lachin JM, Genuth S, Cleary P, Davis MD, Nathan DM (2000). Retinopathy and nephropathy in patients with type I diabetes four years after a trial of intensive therapy. *The New England Journal of Medicine*.

[5] Nathan DM (2003). Sustained effect of intensive treatment of type 1 diabetes mellitus on development and progression of diabetic nephropathy: the Epidemiology of Diabetes Interventions and Complications (EDIC) Study. *Journal of the American Medical Association*.

[6] Nathan DM, Cleary PA, Backlund J-YC (2005). Intensive diabetes treatment and cardiovascular disease in patients with type 1 diabetes. *The New England Journal of Medicine*.

[7] Holman RR, Paul SK, Bethel MA, Matthews DR, Neil HAW (2008). 10-year follow-up of intensive glucose control in type 2 diabetes. *The New England Journal of Medicine*.

[8] Brownlee M, Cerami A, Vlassara H (1988). Advanced glycosylation end products in tissue and the biochemical basis of diabetic complications. *The New England Journal of Medicine*.

[9] Dyer DG, Blackledge JA, Thorpe SR, Baynes JW (1991). Formation of pentosidine during nonenzymatic browning of proteins by glucose: identification of glucose and other carbohydrates as possible precursors of pentosidine in vivo. *Journal of Biological Chemistry*.

[10] Yamagishi S-I, Imaizumi T (2005). Diabetic vascular complications: pathophysiology, biochemical basis and potential therapeutic strategy. *Current Pharmaceutical Design*.

[11] Rahbar S, Figarola JL (2003). Novel inhibitors of advanced glycation endproducts. *Archives of Biochemistry and Biophysics*.

[12] Yamagishi S-I, Takeuchi M, Inagaki Y, Nakamura K, Imaizumi T (2003). Role of advanced glycation end products (AGEs) and their receptor (RAGE) in the pathogenesis of diabetic microangiopathy. *International Journal of Clinical Pharmacology Research*.

[13] Vlassara H, Palace MR (2002). Diabetes and advanced glycation endproducts. *Journal of Internal Medicine*.

[14] Bierhaus A, Hofmann MA, Ziegler R, Nawroth PP (1998). AGEs and their interaction with AGE-receptors in vascular disease and diabetes mellitus. I. The AGE concept. *Cardiovascular Research*.

[15] Wendt T, Bucciarelli L, Qu W (2002). Receptor for advanced glycation endproducts (RAGE) and vascular inflammation: insights into the pathogenesis of macrovascular complications in diabetes. *Current Atherosclerosis Reports*.

[16] Schmidt AM, Stern D (2000). Atherosclerosis and diabetes: the RAGE connection. *Current Atherosclerosis Reports*.

[17] Stitt AW, Bucala R, Vlassara H (1997). Atherogenesis and advanced glycation: promotion, progression, and prevention. *Annals of the New York Academy of Sciences*.

[18] Takenaka K, Yamagishi S-I, Matsui T, Nakamura K, Imaizumi T (2006). Role of advanced glycation end products (AGEs) in thrombogenic abnormalities in diabetes. *Current Neurovascular Research*.

[19] Sato T, Iwaki M, Shimogaito N, Wu X, Yamagishi S-I, Takeuchi M (2006). TAGE (Toxic AGEs) theory in diabetic complications. *Current Molecular Medicine*.

[20] Takeuchi M, Yamagishi S-I (2009). Involvement of toxic AGEs (TAGE) in the pathogenesis of diabetic vascular complications and Alzheimer’s disease. *Journal of Alzheimer’s Disease*.

[21] Takeuchi M, Makita Z (2001). Alternative routes for the formation of immunochemically distinct advanced glycation end-products in vivo. *Current Molecular Medicine*.

[22] Bucala R, Cerami A (1992). Advanced glycosylation: chemistry, biology, and implications for diabetes and aging. *Advances in Pharmacology*.

[23] Vlassara H, Bucala R, Striker L (1994). Pathogenic effects of advanced glycosylation: biochemical, biologic, and clinical implications for diabetes and aging. *Laboratory Investigation*.

[24] Brownlee M (1995). Advanced protein glycosylation in diabetes and aging. *Annual Review of Medicine*.

[25] Monnier VM, Cerami A (1981). Nonenzymatic browning in vivo: possible process for aging of long-lived proteins. *Science*.

[26] Wells-Knecht KJ, Zyzak DV, Litchfield JE, Thorpe SR, Baynes JW (1995). Mechanism of autoxidative glycosylation: identification of glyoxal and arabinose as intermediates in the autoxidative modification of proteins by glucose. *Biochemistry*.

[27] Thornalley PJ (1996). Pharmacology of methylglyoxal: formation, modification of proteins and nucleic acids, and enzymatic detoxification—a role in pathogenesis and antiproliferative chemotherapy. *General Pharmacology*.

[28] Thornalley PJ, Langborg A, Minhas HS (1999). Formation of glyoxal, methylglyoxal and 3-deoxyglucosone in the glycation of proteins by glucose. *Biochemical Journal*.

[29] Takeuchi M, Makita Z, Yanagisawa K, Kameda Y, Koike T (1999). Detection of noncarboxymethyllysine and carboxymethyllysine advanced glycation end products (AGE) in serum of diabetic patients. *Molecular Medicine*.

[30] Takeuchi M, Makita Z, Bucala R, Suzuki T, Koike T, Kameda Y (2000). Immunological evidence that non-carboxymethyllysine advanced glycation end-products are produced from short chain sugars and dicarbonyl compounds in vivo. *Molecular Medicine*.

[31] Takeuchi M, Yanase Y, Matsuura N (2001). Immunological detection of a novel advanced glycation end-product. *Molecular Medicine*.

[32] Yan SD, Chen X, Fu J (1996). RAGE and amyloid-*β* peptide neurotoxicity in Alzheimer’s disease. *Nature*.

[33] Schmidt AM, Yan SD, Yan SF, Stern DM (2000). The biology of the receptor for advanced glycation end products and its ligands. *Biochimica et Biophysica Acta*.

[34] Bucciarelli LG, Wendt T, Rong L (2002). RAGE is a multiligand receptor of the immunoglobulin superfamily: implications for homeostasis and chronic disease. *Cellular and Molecular Life Sciences*.

[35] Hudson BI, Bucciarelli LG, Wendt T (2003). Blockade of receptor for advanced glycation endproducts: a new target for therapeutic intervention in diabetic complications and inflammatory disorders. *Archives of Biochemistry and Biophysics*.

[36] Ramasamy R, Vannucci SJ, Yan SSD, Herold K, Yan SF, Schmidt AM (2005). Advanced glycation end products and RAGE: a common thread in aging, diabetes, neurodegeneration, and inflammation. *Glycobiology*.

[37] Li YM, Mitsuhashi T, Wojciechowicz D (1996). Molecular identity and cellular distribution of advanced glycation endproduct receptors: relationship of p60 to OST-48 and p90 to 80K-H membrane proteins. *Proceedings of the National Academy of Sciences of the United States of America*.

[38] Vlassara H, Li YM, Imani F (1995). Identification of galectin-3 as a high-affinity binding protein for advanced glycation end products (AGE): a new member of the AGE-receptor complex. *Molecular Medicine*.

[39] Ohgami N, Nagai R, Ikemoto M (2002). CD36, serves as a receptor for advanced glycation endproducts (AGE). *Journal of Diabetes and Its Complications*.

[40] El Khoury J, Thomas CA, Loike JD, Hickman SE, Cao L, Silverstein SC (1994). Macrophages adhere to glucose-modified basement membrane collagen IV *via* their scavenger receptors. *Journal of Biological Chemistry*.

[41] Tamura Y, Adachi H, Osuga JI (2003). FEEL-1 and FEEL-2 are endocytic receptors for advanced glycation end products. *Journal of Biological Chemistry*.

[42] Hudson BI, Harja E, Moser B, Schmidt AM (2005). Soluble levels of receptor for advanced glycation endproducts (sRAGE) and coronary artery disease: the next C-reactive protein?. *Arteriosclerosis, Thrombosis, and Vascular Biology*.

[43] Yonekura H, Yamamoto Y, Sakurai S (2003). Novel splice variants of the receptor for advanced glycation end-products expressed in human vascular endothelial cells and pericytes, and their putative roles in diabetes-induced vascular injury. *Biochemical Journal*.

[44] Yamamoto Y, Yonekura H, Watanabe T (2007). Short-chain aldehyde-derived ligands for RAGE and their actions on endothelial cells. *Diabetes Research and Clinical Practice*.

[45] Takeuchi M, Yamagishi S-I (2004). Alternative routes for the formation of glyceraldehyde-derived AGEs (TAGE) in vivo. *Medical Hypotheses*.

[46] Oates PJ (2002). Polyol pathway and diabetic peripheral neuropathy. *International Review of Neurobiology*.

[47] Maekawa K, Tanimoto T, Okada S (2002). Gene expression of enzymes comprising the polyol pathway in various rat tissues determined by the competitive RT-PCR method. *Japanese Journal of Pharmacology*.

[48] Schalkwijk CG, Stehouwer CDA, van Hinsbergh VWM (2004). Fructose-mediated non-enzymatic glycation: sweet coupling or bad modification. *Diabetes/Metabolism Research and Reviews*.

[49] Gaby AR (2005). Adverse effects of dietary fructose. *Alternative Medicine Review*.

[50] Hallfrisch J (1990). Metabolic effects of dietary fructose. *The FASEB Journal*.

[51] Mayes PA (1993). Intermediary metabolism of fructose. *American Journal of Clinical Nutrition*.

[52] L’Esperance FA, James WA, Judson PH (1990). *Ellenberg and Rifkin’s Diabetes Mellitus, Theory and Practice*.

[53] Mandarino LJ (1992). Current hypotheses for the biochemical basis of diabetic retinopathy. *Diabetes Care*.

[54] Frank RN (1991). On the pathogenesis of diabetic retinopathy: a 1990 update. *Ophthalmology*.

[55] Sims DE (1991). Recent advances in pericyte biology—implications for health and disease. *Canadian Journal of Cardiology*.

[56] Herman IM, D’Amore PA (1985). Microvascular pericytes contain muscle and nonmuscle actins. *Journal of Cell Biology*.

[57] Joyce NC, Haire MF, Palade GE (1985). Contractile proteins in pericytes. II. Immunocytochemical evidence for the presence of two isomyosins in graded concentrations. *Journal of Cell Biology*.

[58] Adamis AP, Miller JW, Bernal MT (1994). Increased vascular endothelial growth factor levels in the vitreous of eyes with proliferative diabetic retinopathy. *American Journal of Ophthalmology*.

[59] Aiello LP, Avery RL, Arrigg PG (1994). Vascular endothelial growth factor in ocular fluid of patients with diabetic retinopathy and other retinal disorders. *The New England Journal of Medicine*.

[60] Stitt AW, Li YM, Gardiner TA, Bucala R, Archer DB, Vlassara H (1997). Advanced glycation end products (AGEs) co-localize with AGE receptors in the retinal vasculature of diabetic and of AGE-infused rats. *American Journal of Pathology*.

[61] Sharma NK, Gardiner TA, Archer DB (1985). A morphologic and autoradiographic study of cell death and regeneration in the retinal microvasculature of normal and diabetic rats. *American Journal of Ophthalmology*.

[62] Yamagishi S-I, Amano S, Inagaki Y (2002). Advanced glycation end products-induced apoptosis and overexpression of vascular endothelial growth factor in bovine retinal pericytes. *Biochemical and Biophysical Research Communications*.

[63] Yamagishi S-I, Amano S, Inagaki Y, Okamoto T, Takeuchi M, Makita Z (2002). Beraprost sodium, a prostaglandin I_2_ analogue, protects against advanced glycation end products-induced injury in cultured retinal pericytes. *Molecular Medicine*.

[64] Yamagishi S-I, Takeuchi M, Matsui T, Nakamura K, Imaizumi T, Inoue H (2005). Angiotensin II augments advanced glycation end product-induced pericyte apoptosis through RAGE overexpression. *FEBS Letters*.

[65] Okamoto T, Yamagishi S-I, Inagaki Y (2002). Incadronate disodium inhibits advanced glycation end products-induced angiogenesis in vitro. *Biochemical and Biophysical Research Communications*.

[66] Okamoto T, Yamagishi S-I, Inagaki Y (2002). Angiogenesis induced by advanced glycation end products and its prevention by cerivastatin. *The FASEB Journal*.

[67] Schroder S, Palinski W, Schmid-Schonbein GW (1991). Activated monocytes and granulocytes, capillary nonperfusion, and neovascularization in diabetic retinopathy. *American Journal of Pathology*.

[68] Moore TCB, Moore JE, Kaji Y (2003). The role of advanced glycation end products in retinal microvascular leukostasis. *Investigative Ophthalmology and Visual Science*.

[69] Inagaki Y, Yamagishi S-I, Okamoto T, Takeuchi M, Amano S (2003). Pigment epithelium-derived factor prevents advanced glycation end products-induced monocyte chemoattractant protein-1 production in microvascular endothelial cells by suppressing intracellular reactive oxygen species generation. *Diabetologia*.

[70] Qiao Q, Larsen S, Borch-Johnsen K (2001). Glucose tolerance and cardiovascular mortality: comparison of fasting and 2-hour diagnostic criteria. *Archives of Internal Medicine*.

[71] Levitan EB, Song Y, Ford ES, Liu S (2004). Is nondiabetic hyperglycemia a risk factor for cardiovascular disease? A meta-analysis of prospective studies. *Archives of Internal Medicine*.

[72] Shiraiwa T, Kaneto H, Miyatsuka T (2005). Post-prandial hyperglycemia is an important predictor of the incidence of diabetic microangiopathy in Japanese type 2 diabetic patients. *Biochemical and Biophysical Research Communications*.

[73] Kitahara Y, Takeuchi M, Miura K, Mine T, Matsui T, Yamagishi S-I (2008). Glyceraldehyde-derived advanced glycation end products (AGEs). A novel biomarker of postprandial hyperglycaemia in diabetic rats. *Clinical and Experimental Medicine*.

[74] Du X, Matsumura T, Edelstein D (2003). Inhibition of GAPDH activity by poly(ADP-ribose) polymerase activates three major pathways of hyperglycemic damage in endothelial cells. *Journal of Clinical Investigation*.

[75] Monnier L, Lapinski H, Colette C (2003). Contributions of fasting and postprandial plasma glucose increments to the overall diurnal hyperglycemia of type 2 diabetic patients: variations with increasing levels of HbA. *Diabetes Care*.

[76] Koga K, Yamagishi S-I, Okamoto T (2002). Serum levels of glucose-derived advanced glycation end products are associated with the severity of diabetic retinopathy in type 2 diabetic patients without renal dysfunction. *International Journal of Clinical Pharmacology Research*.

[77] Miura J, Yamagishi S-I, Uchigata Y (2003). Serum levels of non-carboxymethyllysine advanced glycation endproducts are correlated to severity of microvascular complications in patients with type 1 diabetes. *Journal of Diabetes and Its Complications*.

[78] Miura J, Uchigata Y, Yamamoto Y (2004). AGE down-regulation of monocyte RAGE expression and its association with diabetic complications in type 1 diabetes. *Journal of Diabetes and Its Complications*.

[79] Jinnouchi Y, Yamagishi S-I, Takeuchi M (2006). Atorvastatin decreases serum levels of advanced glycation end products (AGEs) in patients with type 2 diabetes. *Clinical and Experimental Medicine*.

[80] Yokoi M, Yamagishi S-I, Takeuchi M (2005). Elevations of AGE and vascular endothelial growth factor with decreased total antioxidant status in the vitreous fluid of diabetic patients with retinopathy. *British Journal of Ophthalmology*.

[81] Yokoi M, Yamagishi S-I, Takeuchi M (2007). Positive correlation between vitreous levels of advanced glycation end products and vascular endothelial growth factor in patients with diabetic retinopathy sufficiently treated with photocoagulation. *British Journal of Ophthalmology*.

[82] Enomoto M, Adachi H, Yamagishi S-I (2006). Positive association of serum levels of advanced glycation end products with thrombogenic markers in humans. *Metabolism*.

[83] Sugiyama S, Miyata T, Ueda Y (1998). Plasma levels of pentosidine in diabetic patients: an advanced glycation end product. *Journal of the American Society of Nephrology*.

[84] Yamaguchi M, Nakamura N, Nakano K (1998). Immunochemical quantification of crossline as a fluorescent advanced glycation endproduct in erythrocyte membrane proteins from diabetic patients with or without retinopathy. *Diabetic Medicine*.

[85] Wagner Z, Wittmann I, Mazak I (2001). N*ε*-(carboxymethyl)lysine levels in patients with type 2 diabetes: role of renal function. *American Journal of Kidney Diseases*.

[86] Park L, Raman KG, Lee KJ (1998). Suppression of accelerated diabetic atherosclerosis by the soluble receptor for advanced glycation endproducts. *Nature Medicine*.

[87] Bucciarelli LG, Wendt T, Qu W (2002). RAGE blockade stabilizes established atherosclerosis in diabetic apolipoprotein E-null mice. *Circulation*.

[88] Barile GR, Pachydaki S-I, Tari SR (2005). The RAGE axis in early diabetic retinopathy. *Investigative Ophthalmology and Visual Science*.

[89] Kaji Y, Usui T, Ishida S (2007). Inhibition of diabetic leukostasis and blood-retinal barrier breakdown with a soluble form of a receptor for advanced glycation end products. *Investigative Ophthalmology and Visual Science*.

[90] Challier M, Jacqueminet S, Benabdesselam O, Grimaldi A, Beaudeux JL (2005). Increased serum concentrations of soluble receptor for advanced glycation endproducts in patients with type 1 diabetes. *Clinical Chemistry*.

[91] Nakamura K, Yamagishi S-I, Adachi H (2007). Elevation of soluble form of receptor for advanced glycation end products (sRAGE) in diabetic subjects with coronary artery disease. *Diabetes/Metabolism Research and Reviews*.

[92] Tan KC, Shiu SW, Chow WS, Leng L, Bucala R, Betteridge DJ (2006). Association between serum levels of soluble receptor for advanced glycation end products and circulating advanced glycation end products in type 2 diabetes. *Diabetologia*.

[93] Nakamura K, Yamagishi S-I, Matsui T, Adachi H, Takeuchi M, Imaizumi T (2007). Serum levels of soluble form of receptor for advanced glycation end products (sRAGE) are correlated with AGEs in both diabetic and non-diabetic subjects. *Clinical and Experimental Medicine*.

[94] Yamagishi S-I, Adachi H, Nakamura K (2006). Positive association between serum levels of advanced glycation end products and the soluble form of receptor for advanced glycation end products in nondiabetic subjects. *Metabolism*.

[95] Nakamura K, Yamagishi S-I, Adachi H (2007). Serum levels of sRAGE, the soluble form of receptor for advanced glycation end products, are associated with inflammatory markers in patients with type 2 diabetes. *Molecular Medicine*.

[96] Nakamura K, Yamagishi S-I, Adachi H (2008). Circulating advanced glycation end products (AGEs) and soluble form of receptor for AGEs (sRAGE) are independent determinants of serum monocyte chemoattractant protein-1 (MCP-1) levels in patients with type 2 diabetes. *Diabetes/Metabolism Research and Reviews*.

[97] Yamagishi S-I, Imaizumi T (2007). Serum levels of soluble form of receptor for advanced glycation end products (sRAGE) may reflect tissue RAGE expression in diabetes. *Arteriosclerosis, Thrombosis, and Vascular Biology*.

[98] Yamagishi S-I, Matsui T, Nakamura K (2007). Kinetics, role and therapeutic implications of endogenous soluble form of receptor for advanced glycation and products (sRAGE) in diabetes. *Current Drug Targets*.

[99] Miura J, Yamamoto Y, Osawa M (2007). Endogenous secretory receptor for advanced glycation endproducts levels are correlated with serum pentosidine and CML in patients with type 1 diabetes. *Arteriosclerosis, Thrombosis, and Vascular Biology*.

[100] Katakami N, Matsuhisa M, Kaneto H (2005). Decreased endogenous secretory advanced glycation end product receptor in type 1 diabetic patients: its possible association with diabetic vascular complications. *Diabetes Care*.

[101] Sakurai S, Yamamoto Y, Tamei H (2006). Development of an ELISA for esRAGE and its application to type 1 diabetic patients. *Diabetes Research and Clinical Practice*.

[102] Koyama H, Shoji T, Yokoyama H (2005). Plasma level of endogenous secretory RAGE is associated with components of the metabolic syndrome and atherosclerosis. *Arteriosclerosis, Thrombosis, and Vascular Biology*.

[103] Humpert PM, Djuric Z, Kopf S (2007). Soluble RAGE but not endogenous secretory RAGE is associated with albuminuria in patients with type 2 diabetes. *Cardiovascular Diabetology*.

[104] Nakamura K, Yamagishi S-I, Nakamura Y (2005). Telmisartan inhibits expression of a receptor for advanced glycation end products (RAGE) in angiotensin-II-exposed endothelial cells and decreases serum levels of soluble RAGE in patients with essential hypertension. *Microvascular Research*.

[105] Mamputu JC, Renier G (2004). Advanced glycation end-products increase monocyte adhesion to retinal endothelial cells through vascular endothelial growth factor-induced ICAM-1 expression: inhibitory effect of antioxidants. *Journal of Leukocyte Biology*.

[106] Miyamoto K, Khosrof S, Bursell SE (1999). Prevention of leukostasis and vascular leakage in streptozotocin-induced diabetic retinopathy *via* intercellular adhesion molecule-1 inhibition. *Proceedings of the National Academy of Sciences of the United States of America*.

[107] Matsumoto Y, Takahashi M, Ogata M (2002). Relationship between glycoxidation and cytokines in the vitreous of eyes with diabetic retinopathy. *Japanese Journal of Ophthalmology*.

[108] Okada S, Shikata K, Matsuda M (2003). Intercellular adhesion molecule-1-deficient mice are resistant against renal injury after induction of diabetes. *Diabetes*.

[109] Chow FY, Nikolic-Paterson DJ, Ozols E, Atkins RC, Rollin BJ, Tesch GH (2006). Monocyte chemoattractant protein-1 promotes the development of diabetic renal injury in streptozotocin-treated mice. *Kidney International*.

[110] De Vriese AS, Tilton RG, Elger M, Stephan CC, Kriz W, Lameire NH (2001). Antibodies against vascular endothelial growth factor improve early renal dysfunction in experimental diabetes. *Journal of the American Society of Nephrology*.

[111] Flyvbjerg A, Dagnæs-Hansen F, De Vriese AS, Schrijvers BF, Tilton RG, Rasch R (2002). Amelioration of long-term renal changes in obese type 2 diabetic mice by a neutralizing vascular endothelial growth factor antibody. *Diabetes*.

[112] Yamagishi S-I, Nakamura K, Takenaka K, Matsui T, Inoue H (2006). Pleiotropic effects of nifedipine on atherosclerosis. *Current Pharmaceutical Design*.

[113] Celletti FL, Waugh JM, Amabile PG, Brendolan A, Hilfiker PR, Dake MD (2001). Vascular endothelial growth factor enhances atherosclerotic plaque progression. *Nature Medicine*.

[114] Yamagishi S-I, Nakamura K, Matsui T (2007). Potential utility of telmisartan, an angiotensin II type 1 receptor blocker with peroxisome proliferator-activated receptor-*γ* (PPAR-*γ*)-modulating activity for the treatment of cardiometabolic disorders. *Current Molecular Medicine*.

[115] Matsui T, Yamagishi S-I, Ueda S (2007). Telmisartan, an angiotensin II type 1 receptor blocker, inhibits advanced glycation end-product (AGE)-induced monocyte chemoattractant protein-1 expression in mesangial cells through downregulation of receptor for AGEs *via* peroxisome proliferator-activated receptor-*γ*, activation. *Journal of International Medical Research*.

[116] Yoshida T, Yamagishi S-I, Nakamura K (2006). Telmisartan inhibits AGE-induced C-reactive protein production through downregulation of the receptor for AGE *via* peroxisome proliferator-activated receptor-gamma activation. *Diabetologia*.

[117] Yamagishi S-I, Matsui T, Nakamura K (2008). Olmesartan blocks advanced glycation end products (AGEs)-induced angiogenesis in vitro by suppressing receptor for AGEs (RAGE) expression. *Microvascular Research*.

[118] Yamagishi S-I, Matsui T, Nakamura K (2007). Olmesartan blocks inflammatory reactions in endothelial cells evoked by advanced glycation end products by suppressing generation of reactive oxygen species. *Ophthalmic Research*.

[119] Tombran-Tink J, Barnstable CJ (2003). PEDF: a multifaceted neurotrophic factor. *Nature Reviews Neuroscience*.

[120] Abe R, Fujita Y, Yamagishi S-I (2007). Angiogenesis and metastasis inhibitors for the treatment of malignant melanoma. *Mini-Reviews in Medicinal Chemistry*.

[121] Yamagishi S-I, Inagaki Y, Amano S, Okamoto T, Takeuchi M, Makita Z (2002). Pigment epithelium-derived factor protects cultured retinal pericytes from advanced glycation end product-induced injury through its antioxidative properties. *Biochemical and Biophysical Research Communications*.

[122] Yamagishi S-I, Amano S, Inagaki Y, Okamoto T, Takeuchi M, Inoue H (2003). Pigment epithelium-derived factor inhibits leptin-induced angiogenesis by suppressing vascular endothelial growth factor gene expression through anti-oxidative properties. *Microvascular Research*.

[123] Yamagishi S-I, Inagaki Y, Nakamura K (2004). Pigment epithelium-derived factor inhibits TNF-*α*-induced interleukin-6 expression in endothelial cells by suppressing NADPH oxidase-mediated reactive oxygen species generation. *Journal of Molecular and Cellular Cardiology*.

[124] Yamagishi S-I, Nakamura K, Ueda S, Kato S, Imaizumi T (2005). Pigment epithelium-derived factor (PEDF) blocks angiotensin II signaling in endothelial cells via suppression of NADPH oxidase: a novel anti-oxidative mechanism of PEDF. *Cell and Tissue Research*.

[125] Yamagishi S-I, Matsui T, Nakamura K (2006). Pigment-epithelium-derived factor (PEDF) inhibits angiotensin-II-induced vascular endothelial growth factor (VEGF) expression in MOLT-3 T cells through anti-oxidative properties. *Microvascular Research*.

[126] Yamagishi S-I, Ueda S, Matsui T (2007). Pigment epithelium-derived factor (PEDF) prevents advanced glycation end products (AGEs)-elicited endothelial nitric oxide synthase (eNOS) reduction through its anti-oxidative properties. *Protein and Peptide Letters*.

[127] Yamagishi S-I, Matsui T, Nakamura K, Takeuchi M, Imaizumi T (2006). Pigment epithelium-derived factor (PEDF) prevents diabetes- or advanced glycation end products (AGE)-elicited retinal leukostasis. *Microvascular Research*.

[128] Yamagishi S-I, Matsui T, Nakamura K (2007). Pigment-epithelium-derived factor suppresses expression of receptor for advanced glycation end products in the eye of diabetic rats. *Ophthalmic Research*.

[129] Yamagishi S-I, Nakamura K, Matsui T (2006). Pigment epithelium-derived factor inhibits advanced glycation end product-induced retinal vascular hyperpermeability by blocking reactive oxygen species-mediated vascular endothelial growth factor expression. *Journal of Biological Chemistry*.

[130] Spranger J, Osterhoff M, Reimann M (2001). Loss of the antiangiogenic pigment epithelium-derived factor in patients with angiogenic eye disease. *Diabetes*.

[131] Boehm BO, Lang G, Volpert O (2003). Low content of the natural ocular anti-angiogenic agent pigment epithelium-derived factor (PEDF) in aqueous humor predicts progression of diabetic retinopathy. *Diabetologia*.

[132] Yokoi M, Yamagishi S-I, Saito A (2007). Positive association of pigment epithelium-derived factor with total antioxidant capacity in the vitreous fluid of patients with proliferative diabetic retinopathy. *British Journal of Ophthalmology*.

[133] Yoshida Y, Yamagishi S-I, Matsui T (2007). Positive correlation of pigment epithelium-derived factor and total antioxidant capacity in aqueous humour of patients with uveitis and proliferative diabetic retinopathy. *British Journal of Ophthalmology*.

[134] Yoshida T, Yamagishi S-I, Nakamura K (2007). Atorvastatin inhibits advanced glycation end products (AGE)-induced C-reactive expression in hepatoma cells by suppressing reactive oxygen species generation. *Vascular Disease Prevention*.

[135] Yamagishi S-I, Matsui T, Nakamura K, Takeuchi M (2005). Minodronate, a nitrogen-containing bisphosphonate, inhibits advanced glycation end product-induced vascular cell adhesion molecule-1 expression in endothelial cells by suppressing reactive oxygen species generation. *International Journal of Tissue Reactions*.

[136] Yamagishi S-I, Nakamura K, Matsui T, Takeuchi M (2005). Minodronate, a nitrogen-containing bisphosphonate, is a promising remedy for treating patients with diabetic retinopathy. *Medical Hypotheses*.

[137] Yamagishi S-I, Abe R, Inagaki Y (2004). Minodronate, a newly developed nitrogen-containing bisphosphonate, suppresses melanoma growth and improves survival in nude mice by blocking vascular endothelial growth factor signaling. *American Journal of Pathology*.

[138] Shamoon H, Duffy H, Fleischer N (1993). The effect of intensive treatment of diabetes on the development and progression of long-term complications in insulin-dependent diabetes mellitus. *The New England Journal of Medicine*.

[139] Turner R (1998). Intensive blood-glucose control with sulphonylureas or insulin compared with conventional treatment and risk of complications in patients with type 2 diabetes (UKPDS 33). *The Lancet*.

